# All-Atom Mesoscale Simulations Predict the Conformational Dynamics of Influenza Virus Surface Glycoproteins

**DOI:** 10.1021/acscentsci.3c00020

**Published:** 2023-01-10

**Authors:** Dheeraj Prakaash, Syma Khalid

**Affiliations:** Department of Biochemistry, University of Oxford, South Parks Road, Oxford OX1 3QU, U.K.

Recent advances in molecular dynamics (MD) simulation methodologies (in particular, where they leverage machine learning algorithms for enhanced/efficient sampling) as well as access to ever increasing amounts of computational resources have meant that molecular simulation of entire viral particles has become tractable. In a recent issue of *ACS Central Science*, Amaro and co-workers characterize the dynamics of the two surface glycoproteins of influenza A using atomistic simulations of virion envelope models.^1^

The influenza virus is ∼80–100 nm in diameter. It is surrounded by a membrane comprising a phospholipid bilayer with three major embedded proteins, the M2 proton channel and the glycoproteins, hemagglutinin (HA) and neuraminidase (NA). The glycoproteins extend from the surface of the membrane into the external environment. Owing largely to its obvious biomedical importance and the almost constant need for development of new therapeutics that target it, the influenza virus has been the focus of several molecular simulation studies. While mechanistic details of individual influenza proteins as well as isolated domains (e.g., the transmembrane domain of the M2 protein) have been gleaned from simulation studies often conducted alongside complementary experimental methods,^2−4^ larger systems incorporating multiple protein copy numbers have been relatively recent, for example, coarse-grained simulations from Sansom and co-workers and Voth and co-workers.^5,6^ A more recent study by the Amaro group employed atomistic simulations to study a model of the virion envelope.^7^ In all these cases, the surface protein glycans were not incorporated into the models.

Building upon the aforementioned earlier work of their own group, Amaro and co-workers report a molecular simulation study in which the dynamics of the two influenza surface glycoproteins HA and NA are identified and quantified within models of the envelopes of two evolutionarily linked strains of influenza A, namely, A/swine/Shandong/N1/2009(H1N1) (H1N1-Shan2009) and A/45/Michigan/2015(H1N1) (H1N1-Mich2015) (PMID: 35982676). Thus, they provide the first atomistic-resolution insights into the dynamics of these glycoproteins within a realistically crowded membrane environment in which the glycosylation of the proteins is incorporated. The virion envelope model was composed of 3-palmitoyl-2-oleoyl-dglycero-1-phosphatidylcholine (POPC) lipids in a quasi-spherical lipid bilayer, in which 236 HA trimers, 30 NA tetramers, and 11 M2 ion channels were embedded. Crucially, both HA and NA proteins were glycosylated with N-linked glycans, giving a total system size of ∼160 million atoms. Despite the compositional complexity and large system size, the authors employed all-atom (AA) simulations resolution using the Oak Ridge National Lab Titan and NSF Blue Waters supercomputers, which enabled consideration of fine-grained details otherwise inaccessible with the computationally less demanding, but lower resolution coarse-grained models alone.

The authors combined AAMD simulations with Markov state models (MSM) to study the dynamical behavior of the glycoproteins. This approach revealed both proteins to be remarkably flexible and enabled identification and kinetic characterization of their major conformational motions. These can be summarized as (1) NA head tilting, (2) HA ectodomain tilting, and (3) HA head breathing. Two atomistic simulations, one of each strain (H1N1-Shan2009 simulated for 442 ns and H1N1-Mich2015 simulated for 425 ns) were performed, giving extensive sampling of each protein (each one is present in the virion envelope model in multiple copy numbers) from which a two-state MSM was constructed for kinetic analysis of each motion. The impact of the individual motions as well as the synergistic interactions between them, in the context of normal functioning of the virus as well as potential routes to therapeutic targeting of the virus, are discussed.

**Figure 1:**
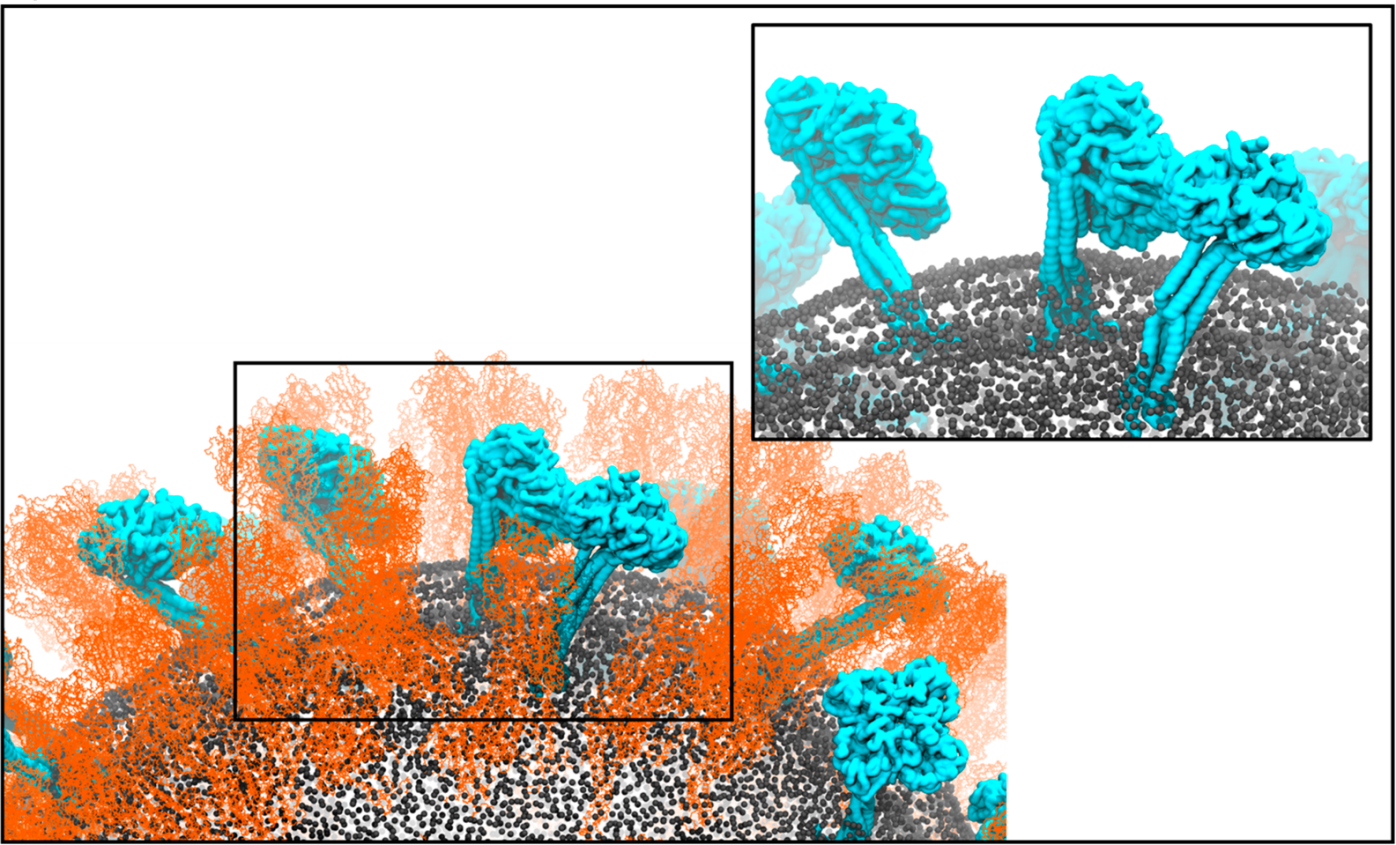
A portion of the model H1N1-Mich2015 virion membrane after 425 ns of simulation. Hemagglutinin (HA) is shown in orange, neuraminidase (NA) are in cyan, and the phosphorus atoms of the lipids are shown as gray spheres. A selection from within the range of NA tilting angles can clearly be seen.

The architecture of NA is that of a propeller-shaped globular head region located on top of a long “stalk”, and the functional unit is thought to be a tetramer. MD simulations revealed the propensity of the head to undergo a range of extensive tilting motions (over 90° relative to the stalk axis), giving the protein remarkable flexibility (Figure 1). MSM were used to characterize the transitions between the tilted and untilted states and enabled comparison between the two strains. A particularly impressive feature of this study is the attention to the immunological implications of the conformational behaviors identified from the simulations. Here it was shown that a human monoclonal antibody, termed NDS.1, recognizes and binds (with 10^–8^ M affinity) the NA head region of two distinct NA tetramers. 3D reconstruction from negative-stain electron microscopy (NS-EM) revealed that NDS.1 Fab recognizes an epitope located at the underside of the NA head. Crucially, this epitope only becomes directly accessible to the antibody once the NA head tilts in the manner revealed by the simulations.

**Figure 2:**
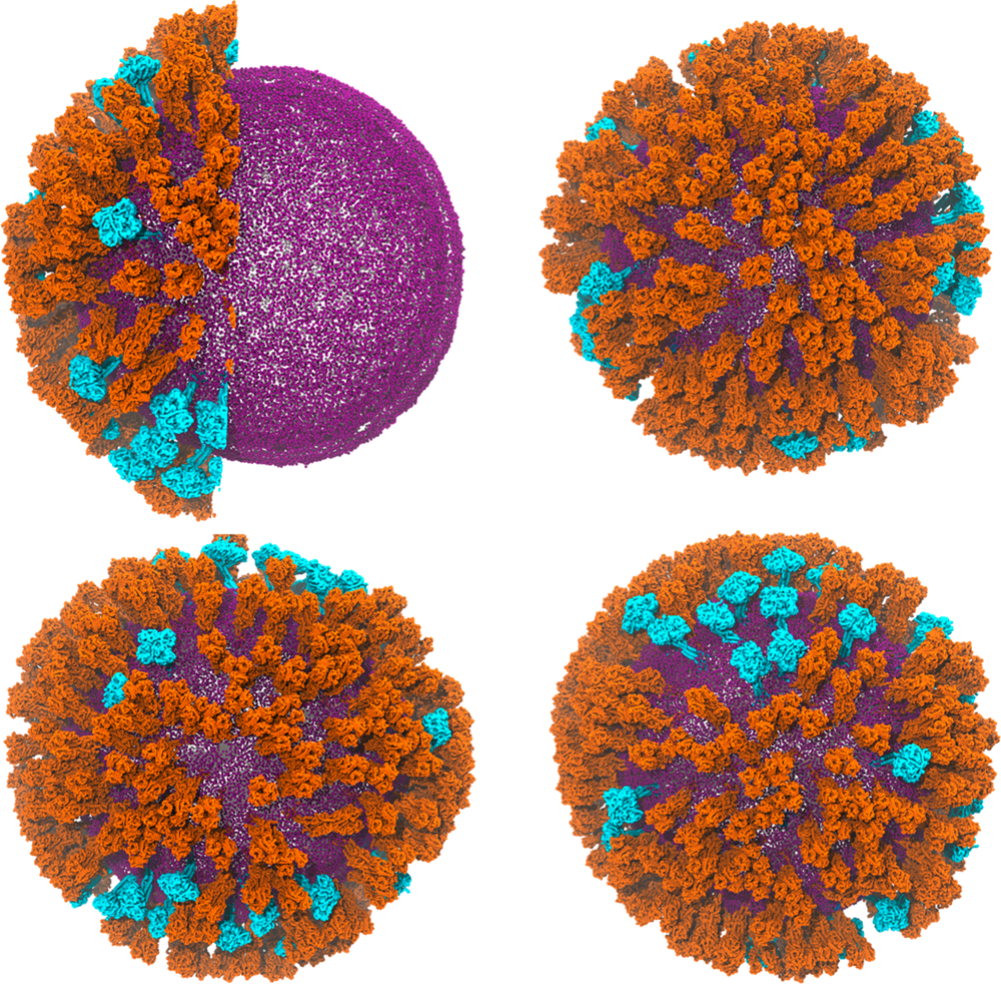
Four different views of the surface of model H1N1-Mich2015 membrane after 425 ns of simulation. In the top left panel, the sphericity of the vesicle can be seen. The proteins are colored as Figure 1, but here the lipid phosphorus atoms are shown in purple. Clustering of the proteins is seen in all four views.

The crowded virion envelope models also enabled the authors to consider the dynamic interplay between the surface glycoproteins. The glycoproteins were not observed to diffuse across the model viral membrane to any great extent but rather to interact with each other through their flexible extra-virion functional domains. Detailed analyses of this interplay revealed the importance of the glycans in forming glycoprotein-glycoprotein interactions.

A key feature of the Amaro study is the use of experimental data wherever possible, for example, in setting up the virion envelope, comparison of simulated protein conformations with structural data, and docking of antibody structures to the glycoproteins. This anchoring of simulations to experimental data enables the authors to place their work in a relevant biomedical context. They demonstrate the importance of including realistic crowding and glycans when considering the dynamics of viral glycoproteins. Finally, they elegantly make use of the multiple protein copy numbers for enhanced sampling of each glycoprotein type from single relatively short simulations, to enable kinetic analyses via MSM. As we have learned from the recent COVID-19 pandemic, molecular simulations can play a key role in understanding the fundamental mechanisms of virus dynamics and infection. The translation of this knowledge promises to facilitate the rational development of novel therapeutics for a range of viral threats, including those for which vaccines are currently being developed, e.g., human immunodeficiency virus (HIV) and Ebola.
